# Quacks

**Published:** 1875-12

**Authors:** 


QUACKS.—From week to week we are in receipt of letters from the afflicted, or their friends, making inquries in reference to the various quacks and quack establishments which infest our city. These letters are in some instances from physicians, who write in the interest of their patients. Physicians, if they will reflect for a moment, must know that the advertisements which flood the country, in the form of posters, newspaper puffs, handbills, etc., etc., emanating from individuals or establishments, either from this city or any other point, are not from the regular physicians, but from adventurers, who prey upon poor suffers who are sufficiently credulous to be caught by their many pledges. The true physicians resort to no such means. If the people could only know the fact that such advertisements are positive evidence of the quack, much suffering, physically, mentally and pecuniarily, would be prevented.

